# Supplementary material for: A transcriptomics resource reveals a transcriptional transition during ordered sarcomere morphogenesis in flight muscle
Source: eLife. 2018 May 30;7:e34058. doi: 10.7554/eLife.34058 (PMC6005683; doi:10.7554/eLife.34058)
Supplement: Transparent reporting form [file elife-34058-transrepform.docx]

***eLife’s* transparent reporting form**

We encourage authors to provide detailed information *within their submission* to facilitate the interpretation and replication of experiments. Authors can upload supporting documentation to indicate the use of appropriate reporting guidelines for health-related research (see [EQUATOR Network](http://www.equator-network.org/%20)), life science research (see the [BioSharing Information Resource](https://biosharing.org/)), or the [ARRIVE guidelines](http://www.plosbiology.org/article/info:doi/10.1371/journal.pbio.1000412) for reporting work involving animal research. Where applicable, authors should refer to any relevant reporting standards documents in this form.

If you have any questions, please consult our Journal Policies and/or contact us: [editorial@elifesciences.org](mailto:editorial@elifesciences.org).

**Sample-size estimation**

- You should state whether an appropriate sample size was computed when the study was being designed
- You should state the statistical method of sample size computation and any required assumptions
- If no explicit power analysis was used, you should describe how you decided what sample (replicate) size (number) to use

Please outline where this information can be found within the submission (e.g., sections or figure legends), or explain why this information doesn’t apply to your submission:

RNA-SEQ was done in duplicates or triplicates as listed in the Material and Methods section and in Figure 4.

Sample sizes for all plots are included in Supplementary File 5. Sample sizes for experiments are additionally listed in the Materials and Methods section or in the Figures or figure legends.

For example, quantitative live imaging was done with at least 10 flies, as listed in the Material and Methods section and Supplementary File 5.

Flight tests were done with more than 80 flies as shown in Figure 7 S1. For sarcomere and myofiber length measurements at least 10 different animals were used as listed in the Material and Methods section.

For myofibril diameter quantifications at least 20 cross sections from more than 10 flies were used as stated in the Materials and Method section.

**Replicates**

- You should report how often each experiment was performed
- You should include a definition of biological versus technical replication
- The data obtained should be provided and sufficient information should be provided to indicate the number of independent biological and/or technical replicates
- If you encountered any outliers, you should describe how these were handled
- Criteria for exclusion/inclusion of data should be clearly stated
- High-throughput sequence data should be uploaded before submission, with a private link for reviewers provided (these are available from both GEO and ArrayExpress)

Please outline where this information can be found within the submission (e.g., sections or figure legends), or explain why this information doesn’t apply to your submission:

mRNA-sequencing was performed in biological duplicate or triplicate, based on financial constraints and the time required to prepare samples, as reported in the Materials and Methods. 100-200 individual flies were dissected per sample, making biological variability between samples minimal.

Replicate data for all plots and calculations are included in Supplementary File 5. This table lists the number of animals, number of images and number of experimental replicates for data presented in Figures 1, 5, 6, 7 and 8.

**Statistical reporting**

- Statistical analysis methods should be described and justified
- Raw data should be presented in figures whenever informative to do so (typically when N per group is less than 10)
- For each experiment, you should identify the statistical tests used, exact values of N, definitions of center, methods of multiple test correction, and dispersion and precision measures (e.g., mean, median, SD, SEM, confidence intervals; and, for the major substantive results, a measure of effect size (e.g., Pearson's r, Cohen's d)
- Report exact p-values wherever possible alongside the summary statistics and 95% confidence intervals. These should be reported for all key questions and not only when the p-value is less than 0.05.

Please outline where this information can be found within the submission (e.g., sections or figure legends), or explain why this information doesn’t apply to your submission:

Raw counts values from mRNA-Seq experiments are included in Supplementary File 1 and 4. Original BAM files are available from the GEO submission. These tables additionally include statistics and significance values from DESeq2 differential expression analysis.

For data presented in Figures 1, 5, 6, 7 and 8, all relevant statistical data can be found in Supplementary File 5. This includes analysis methods, software used, summary statistics, ANOVA results and multiple comparisons tests, include statistic and exact p-values. Raw data values can be found in the associated Source File files for each figure.

(For large datasets, or papers with a very large number of statistical tests, you may upload a single table file with tests, Ns, etc., with reference to sections in the manuscript.)

**Group allocation**

- Indicate how samples were allocated into experimental groups (in the case of clinical studies, please specify allocation to treatment method); if randomization was used, please also state if restricted randomization was applied
- Indicate if masking was used during group allocation, data collection and/or data analysis

Please outline where this information can be found within the submission (e.g., sections or figure legends), or explain why this information doesn’t apply to your submission:

Samples were allocated into experimental groups by genotype, as stated in the Figure Legends. Experimental groups were defined as wild-type control versus mutant/RNAi condition, or by time point as noted in the Figure Legends and Materials and Methods for Figures 2, 3, 4, 5, 6, 7 and 8.

**Additional data files (“source data”)**

- We encourage you to upload relevant additional data files, such as numerical data that are represented as a graph in a figure, or as a summary table
- Where provided, these should be in the most useful format, and they can be uploaded as “Source data” files linked to a main figure or table
- Include model definition files including the full list of parameters used
- Include code used for data analysis (e.g., R, MatLab)
- Avoid stating that data files are “available upon request”

Please indicate the figures or tables for which source data files have been provided:

Raw numerical values have been provided as source data for the tables presented in Figures 1, 5, 6, 7, 8. Raw data for bioinformatics analysis have been included in Supplementary File 1, 2 and 4, and are part of the GEO submission. The code for the MyofibrilJ software is freely available as stated under Data Availability. All software packages used for the R analysis are listed in Materials and Methods and in the Key Resources Table, with relevant citations.
